# Mechanical Support Escalation to Bridge Anemic Jehovah’s Witness to Cardiac Transplantation

**DOI:** 10.3390/jcm14207296

**Published:** 2025-10-16

**Authors:** Shanon Quach, Yevgeniy Khariton, Jaime Hernandez-Montfort, Jerry Fan

**Affiliations:** 1 Baylor Scott and White Health—Temple, Internal Medicine, Temple, TX 76504, USA; shanon.quach@bswhealth.org; 2 Baylor Scott and White Health—Temple, Cardiology, Temple, TX 76504, USA; yevgeniy.khariton@bswhealth.org (Y.K.); jaime.hernandezmontfort@bswhealth.org (J.H.-M.)

**Keywords:** high-profile transvalvular pump, mechanical circulatory support, Jehovah’s witness, orthotopic heart transplant

## Abstract

**Background**: Jehovah’s Witness (JW) patients pose a unique challenge to cardiac surgery due to their refusal of blood products, typically precluding them from becoming candidates for orthotopic heart transplantation (OHT). While “bloodless” cardiac surgery has been described in ideal candidates, anemic or other hematologic-risk patients are typically excluded. We describe a successful “bloodless” OHT in a non-ideal JW patient with anemia and cardiogenic shock, with ventricular assist through a transvalvular pump to bridge and optimize hematologic status prior to operation. **Case Presentation**: A 58-year-old male JW with end-stage non-ischemic cardiomyopathy (NICM) and an ejection fraction of 15–20% experienced repeated decompensation despite maximal medical therapy and implantable cardioverter–defibrillator (ICD) implantation. Two years since first presentation, he developed cardiogenic shock and required intra-aortic balloon pump (IABP) support. Iatrogenic anemia occurred during IABP placement and required femoral re-access and upgrade to Impella^®^ 5.5 support. During mechanical support, he was given a total blood conservation plan that included intravenous iron, darbepoetin alfa, restricted phlebotomy, and nutritional supplementation. Hemoglobin was increased from 7.8 to 10.4 g/dL. Successful “bloodless” OHT was subsequently performed on him with an estimated blood loss of 200 mL, 72 min cardiopulmonary bypass duration, and no transfusion. He was discharged on the nineth day after surgery with a hemoglobin level of 9.9 g/dL and returned to full activity. **Discussion**: Despite inherent risks, bloodless OHT may safely be performed in selected JW patients by means of multidisciplinary coordination, modern mechanical circulatory support, and hematopoietic stimulation. Our case highlights the utility of Impella^®^ 5.5 as a bridge-to-transplant strategy for anemic, hemodynamically unstable JW patients. This is in harmony with evidence from previous studies indicating similar results for JW and non-JW transplant recipients under strict optimization protocols. It also supports the expansion of candidacy criteria if appropriate planning and modern blood conservation strategies are employed. **Conclusions**: Transfusion religious objection ought not preclude JW patients from lifesaving OHT. With judicious perioperative planning, third-generation transvalvular pumps, and hematologic optimization, “bloodless” heart transplantation is possible—potentially even in non-ideal candidates.

## 1. Introduction

Jehovah’s Witnesses (JWs), a global religious denomination with over 8 million members, are known for their doctrinal resistance to blood transfusion, even of red blood cells, white cells, platelets, and plasma [1]. The faith, based on a biblical interpretation of Acts 15:28–29, presents difficult clinical issues, particularly when impending blood loss is anticipated or when transfusion is the accepted standard of care. Among the most demanding of surgical procedures is cardiac surgery—most notably orthotopic heart transplantation—where perioperative transfusion rates remain high [2].

Certain fractions of blood products, such as albumin, immunoglobulins, and clotting factor concentrates, are permissible to some JW patients, but decisions on their use are highly individualized and open to personal conscience [1]. This necessitates considerable preoperative counseling and joint decision-making with the patient, their family, and ethics committees. The refusal of blood products imposes additional clinical, ethical, and logistical burdens on surgical and anesthetic teams who must find innovative ways to optimize oxygen delivery, minimize blood loss, and enhance endogenous erythropoiesis in this population.

Over the past two decades, advances in Patient Blood Management (PBM)—a multidisciplinary, evidence-based approach to minimizing transfusion—have revolutionized care for JW patients. PBM covers preoperative anemia correction (e.g., iron, vitamin B12, folate, and erythropoiesis-stimulating agents (ESAs)), intraoperative blood conserving techniques (e.g., controlled hypotension, cell salvage, and antifibrinolytics), and postoperative strategies to reduce iatrogenic blood loss and promote recovery [3,4]. PBM aligns with religious objection while benefiting all patients by avoiding short-term morbidity-related transfusion adverse events such as immunologic reactions, transfusion-related acute lung injury (TRALI), infections, and increased long-term mortality [3,4,5,6].

It has been demonstrated that JW patients undergoing complex cardiac surgery, i.e., valve replacement and CABG, produce identical short- and long-term outcomes as transfused matched controls, provided rigorous PBM protocols are utilized [2,4,6]. In fact, outcomes in high-volume centers with the implementation of bloodless technology suggest that transfusion might not be necessary even in risky cardiac procedures. For instance, Chambault et al. reported no operative mortality, stroke, or renal failure differences in JWs undergoing open-heart surgery compared with matched controls [6].

Orthotopic heart transplantation (OHT) is, however, a highly risky setting, where transfusion is still used in up to 90% of patients due to the nature of cardiopulmonary bypass (CPB), surgical bleeding, and preoperative anemia [7]. Despite all of these issues, bloodless successful heart transplantation has been documented in the literature. Mechanical circulatory support (MCS) has been increasingly reported as both a bridge to recovery and transplantation in JW patients. Successful use of veno-arterial ECMO and Impella 5.5 with adjunctive hematopoietic stimulation has been described, demonstrating maintenance of hemoglobin and avoidance of transfusions during prolonged support [8,9]. Similarly, LVAD implantation and HeartMate 3 explantation with orthotopic transplant have been reported without transfusion, utilizing minimally invasive techniques and blood conservation protocols [10]. Dallas et al. reported a time-honored case of bloodless OHT in a JW patient based on a combination of preoperative optimization, intraoperative cell salvage, normovolemic hemodilution, and pharmacologic agents [11]. These cases highlight the importance of tailoring device choice and surgical approach to minimize blood loss.

Herein, we report the multi-dimensional clinical course of a 58-year-old Jehovah’s Witness patient with end-stage NICM who decompensated to cardiogenic shock supported by Impella^®^ and ultimately underwent successful bloodless orthotopic heart transplantation following aggressive hematologic and perioperative optimization. The case illustrates the achievability of sophisticated PBM strategies in critically ill transplant recipients and identifies the benefits of customized, ethically guided, multidisciplinary treatment.

## 2. Case Description

A 58-year-old Jehovah’s Witness male with uncontrolled hypertension presented with a 3-month history of dyspnea, orthopnea, cough, and peripheral edema, consistent with decompensated heart failure. Transthoracic echocardiography showed a left ventricular ejection fraction (LVEF) of 15–20%. Coronary angiography excluded ischemic disease, and cardiac magnetic resonance imaging (cMRI) ruled out infiltrative or inflammatory cardiomyopathy. Genetic testing revealed a desmocollin-2 (DSC2) variant of uncertain significance, and the patient was diagnosed with NICM. He was treated with maximally tolerated guideline-directed medical therapy (empagliflozin, sacubitril–valsartan, metoprolol, and spironolactone) and underwent single-chamber ICD placement for primary prevention of ventricular arrhythmias. Despite therapy, he was repeatedly hospitalized for acute decompensated heart failure, with right heart catheterizations showing low cardiac output and elevated filling pressures.

**Figure 1:**
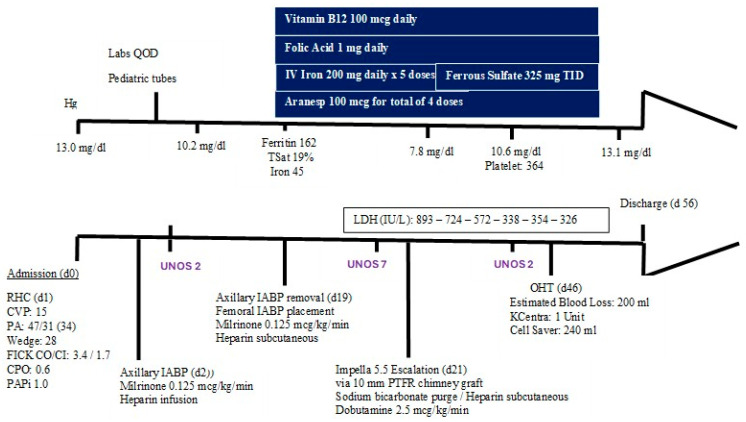
Inpatient Management Timeline.

Approximately two years later, he was admitted in cardiogenic shock (SCAI Stage C). An intra-aortic balloon pump (IABP, Maquet, Wayne, NJ, USA) placed via the left axillary artery provided temporary support but caused access-site bleeding, resulting in iatrogenic anemia (hemoglobin 13 to 10 g/dL). The device was switched to femoral access, but cardiac output remained suboptimal despite inotropic and mechanical support (Figure 1). With ongoing clinical decline and refusal of blood products, escalation to a high-profile transvalvular axial-flow pump (Impella^®^ 5.5, Abiomed, Danvers, MA, USA) was performed, providing more effective circulatory support and stabilizing the patient for further optimization.

While on Impella^®^ support, an intensive hematology optimization strategy was performed. This included intravenous ferric carboxymaltose, darbepoetin alfa for stimulation of erythropoiesis, phlebotomy reduction, and nutrition counseling (Figure 1). A multifaceted approach was utilized to ensure maximal improvement of anemia. Iron studies, folic acid, and vitamin B12 were all tested, which revealed iron deficiency anemia likely from acute blood loss. All essential components of blood production were maintained with oral or intravenous replenishment with standard dosing. Specifically for iron supplementation, ferric carboxymaltose was given daily at five doses and then transitioned to oral ferrous sulfate three times daily. Labs were performed every other day, drawn in pediatric tubes to minimize the volume of blood loss. In addition, darbepoetin alfa targets increasing production, especially in patients with renal dysfunction where erythropoietin might but reduced. The dosing for darbepoetin alfa depended on improvement in hemoglobin by >1 g/dL in 4 weeks [12]. Starting at darbepoetin alfa 100 mcg every 2 weeks, the patient did not have an appropriate response and was switched to 200 mcg every 2 weeks and then progressed to 200 mcg weekly. Although there is concern regarding bleeding, prophylactic anticoagulation should be given with the use of ESAs due to increased risk of thrombosis and stroke [13]. The patient received dietary supplements with meals daily and physical therapy to maintain physical optimization for surgery. We must note that the patient was a motivated individual who ensured that he was meeting his caloric intake, activity, and medication adherence. His hemoglobin nadir was 7.8 g/dL, but improved to 10.4 g/dL preoperatively.

Following multidisciplinary discussion and ethical clearance, the patient underwent uneventful bloodless orthotopic heart transplantation. The procedure was completed with 72 min of cardiopulmonary bypass time and 47 min of aortic cross-clamp time. The protamine dose was based on titration of a point-of-care heparin assay to assure adequate reversal of systemic anticoagulation rather than fixed standard dosing. A thromboelastograph was performed, and prothrombin complex concentrate (K-centra) was given to minimize coagulopathic bleeding. Intraoperative blood loss was minimal (estimated 200 mL). The postoperative period was uneventful, and he was discharged home on postoperative day 9 with a hemoglobin level of 9.9 g/dL. On follow-up, he reported baseline functional status return and continues to be closely monitored in the transplant clinic.

## 3. Discussion

### 3.1. Diagnostic Uncertainty and Relevance to Advanced Heart Failure Management

Although our patient’s genetic testing revealed a desmocollin-2 (DSC2) variant of uncertain significance, the absence of proven pathogenicity limited its diagnostic value. This underscores a common challenge in NICM, where patients may carry variants of unclear clinical significance yet still progress to advanced heart failure. Rather than altering management, such findings highlight the importance of optimizing hemodynamics and candidacy for advanced therapies irrespective of etiology.

For JW patients, this progression to end-stage heart failure has added complexity. Their refusal of transfusions magnifies the risks of decompensation, hospitalization, and invasive interventions. In this context, the focus shifts from diagnostic classification to strategies that can safely bridge unstable, anemic patients to transplantation. Our case emphasizes that while genetic and imaging evaluations help exclude reversible causes, the decisive factor for outcome was timely escalation of mechanical support paired with aggressive PBM, which enabled candidacy for transfusion-free transplantation.

### 3.2. Hemodynamic Decompensation and Mechanical Circulatory Support

At two years of disease duration, the patient demonstrated SCAI Stage C cardiogenic shock with hypotension, end-organ hypoperfusion, and the need for inotropic and mechanical support [14]. Intra-aortic balloon pump (IABP) via the axillary artery, a favored vascular access in ambulatory bridge-to-transplant patients, was attempted with transient stabilization. Bleeding risk related to vascular access is a critical consideration in JW patients, as even modest blood loss may result in clinically significant anemia with limited therapeutic options. The axillary artery is often favored for intra-aortic balloon pump (IABP) placement in ambulatory bridge-to-transplant patients because it allows mobilization and longer support. However, axillary access carries a higher risk of vascular injury and bleeding complications compared with femoral placement. In our case, access-site bleeding during axillary insertion directly resulted in iatrogenic anemia, lowering hemoglobin from 13 g/dL to 10 g/dL. This complication illustrates how access-site selection can profoundly impact management in transfusion-restricted patients, where the burden of anemia not only increases immediate hemodynamic compromise but also narrows the margin of safety for subsequent transplant candidacy. Switching to femoral access stabilized bleeding risk but underscored the importance of anticipating and minimizing vascular complications in this population.

As a precaution against deteriorating hemodynamics and religious prohibition against transfusion, the team augmented support with a transvalvular axial-flow pump (Impella^®^ 5.5)—delivering augmented circulatory support. This action identifies the requirement for prompt decision-making in JW patients, in which every bleeding event can proportionally lead to worse consequences due to limited therapeutic options. Recent case series highlight that MCS can be safely employed in JWs despite their elevated transfusion risk. Reports of Impella^®^ 5.5 and ECMO support in JWs with cardiogenic shock demonstrate that, when paired with hematopoietic stimulation and meticulous conservation strategies, these devices can stabilize hemodynamics while avoiding transfusions [8,9]. Our case extends this literature by demonstrating not just single-device use, but stepwise escalation of MCS to successfully bridge to transplant.

### 3.3. Patient Blood Management: A Multimodal Imperative

The foundation of success in this case was a well-disciplined, interdisciplined PBM protocol initiated with Impella^®^ implantation. The goals were threefold: (1) enhance erythropoiesis, (2) reduce iatrogenic blood loss, and (3) maximize oxygen delivery per unit of circulating hemoglobin. He was optimized with intravenous iron (ferric carboxymaltose) to rapidly replenish iron stores. Darbepoetin alfa, an extended half-life ESA, was used to supplement endogenous red blood cell production. While hospitalized, pediatric tubes and point-of-care testing to minimize diagnostic blood loss. We were also able to optimize dietary intake with sufficient folate, B12, and protein intake. Over the course of several days, the patient’s hemoglobin increased from a low of 7.8 g/dL to 10.4 g/dL prior to transplant—a substantial increase despite ongoing mechanical support and without transfusion. Of special note, transfusion thresholds for the operating room during cardiac surgery are typically >8 g/dL; thus, this level of preoperative increase provided ample room for safety [5].

The efficacy of this approach is attested by numerous studies. Goodnough et al. demonstrated that preoperative ESA and IV iron therapy substantially reduced transfusion need in orthopedic and cardiac surgery [4]. Klein et al. guidelines recommend the same interventions for JW patients, along with antifibrinolytics and meticulous surgical technique [5].

Comparative studies now show that with rigorous PBM, outcomes in JW patients undergoing heart transplantation are equivalent to those in non-JW patients. In a Cedars-Sinai case–control series, JW recipients had similar rates of graft dysfunction, rejection, vasculopathy, and survival compared to matched controls [15]. Likewise, single-center protocols targeting preoperative hemoglobin ≥ 12 g/dL have demonstrated significant reductions in adverse outcomes, reinforcing the centrality of pre-transplant optimization [16].

### 3.4. Intraoperative Strategies and Surgical Success

While optimization in the pre-operative phase can often lead to successful surgical intervention, many considerations for salvaging blood products should also be considered. Cell salvage, which reinfuses and collects intraoperatively lost autologous blood, can be a helpful technique and often acceptable to JW patients when connected continuously to the patient. Aortic cross-clamping and CPB minimization can also be important in reducing blood loss; in this case, a cross-clamp time of 47 min and a total bypass time of 72 min allowed for rapid cardiac transplantation with minimal blood loss. Intraoperative administration of prothrombin complex concentrate 523 units and protamine 20 mg aimed to reduce blood requirements and reverse the use of unfractionated heparin during cardiopulmonary bypass. It has been shown that using protamine–heparin assays to titrate the specific dose for anticoagulation reversal is more effective than the fixed ratio of 1 mg protamine for 100 units of previously administered heparin [17]. There are also multiple algorithms standardizing viscoelastic testing to guide transfusion intraoperatively to minimize bleeding and use of blood products [18]. In total, the estimated blood loss of 200 mL, with evidence of careful technique and optimal coagulopathy management. There was no blood or component therapy used. The procedure progressed well, and postoperative hemoglobin stabilized at 9.9 g/dL.

Recent large-scale evidence further supports these intraoperative strategies. In a cohort of 329 Jehovah’s Witness patients, careful extracorporeal circulation (ECC) management—particularly adjustments to circuit priming, strict hemodilution thresholds, and calculated hemoglobin targets—allowed mean postoperative hemoglobin to remain above 11 g/dL, with low morbidity and favorable survival [19]. An accompanying editorial highlighted the value of detailed intraoperative planning, including oxygen delivery calculations and hemoglobin trajectory modeling, to safeguard against anemia in transfusion-restricted patients [20]. Our case reinforces these findings, showing that when such principles are applied, safe transplantation is possible even in high-risk, anemic patients.

Similar success has been reported in smaller series and case reports. Dallas et al. described maintaining hemoglobin concentrations above 11 g/dL in a JW transplant recipient using a multimodal conservation protocol, and institutional programs such as the Valdecilla group in Spain have demonstrated reproducible outcomes with structured blood management protocols [11,21]. More recently, Tsukioka et al. reported a successful bloodless OHT in a patient with complex congenital anatomy, emphasizing the need for careful surgical planning [22]. Together with our experience, these cases illustrate that advanced techniques can make even high-risk or anatomically challenging patients viable transplant candidates.

### 3.5. Wider Implications and Ethical Aspects

An additional ethical dimension relates to organ allocation. Given the persistent shortage of donor hearts, the medical profession carries a responsibility to ensure transplantation in candidates with the highest likelihood of success and minimized risk of graft failure due to patient-related factors. Historically, this consideration contributed to the exclusion of Jehovah’s Witnesses from candidacy, as refusal of transfusion was perceived as increasing procedural risk. However, emerging evidence demonstrates that with rigorous patient blood management and multidisciplinary planning, outcomes for Jehovah’s Witnesses can be comparable to those of transfusion-eligible patients [15,23]. In addition, a 2025 scoping review of transfusion-free organ transplantation demonstrated that solid organ transplants, including kidney, liver, heart, and lung, can be performed successfully without blood products when patients are carefully prepared and managed by multidisciplinary teams [24]. Thus, the obligation to steward scarce donor organs must be balanced against respect for patient autonomy and the growing evidence base supporting transfusion-free transplantation. Our case further challenges outdated restrictions by showing that even anemic and hemodynamically unstable JW patients can be successfully bridged and transplanted.

This case proves that bloodless transplantation can be achieved—even in severely ill patients—when the protocol-based PBM is paired with robust multidisciplinary collaboration. The care team will also have to anticipate complications, communicate candidly, and involve bioethics teams early on, especially when standard-of-care therapies (such as transfusion) are declined.

It is worth mentioning that this model can be used beyond religious populations. From studies, it has been seen that the proper use of blood in conformity with PBM principles improves the outcomes of the general population. For example, Shander et al. highlighted that PBM reduces infection risk, hospital stay, and mortality [3].

Ethically, patient autonomy remains the central philosophy of modern medicine. The case highlights that religious convictions can be honored without compromising clinical excellence—if institutions are willing to adapt, innovate, and collaborate.

Future avenues for research include the development of standardized PBM protocols tailored to MCS-supported JW patients, comparative studies of transfusion-free versus transfusion-eligible transplant cohorts, and investigation into novel hematopoietic or antifibrinolytic therapies during prolonged MCS bridging.

## 4. Conclusions

This case describes the successful application of multi-faceted patient blood management to enable bloodless orthotopic heart transplantation in a Jehovah’s Witness patient who had severe heart failure and cardiogenic shock on mechanical circulatory support. Through the administration of a systematic, evidence-based, and ethically sound PBM program—erythropoietin-stimulating agents, intravenous iron, limited phlebotomy, and intraoperative conservation strategies—the transplantation was safely performed without allogeneic transfusion. Our experience shows the need for early planning, multidisciplinary working, and individualized planning.

Our findings align with and extend the existing literature demonstrating that transfusion-free transplantation is feasible and safe in Jehovah’s Witnesses when multidisciplinary PBM is rigorously applied. Importantly, this case suggests that candidacy criteria for heart transplantation in JW patients can be broadened, even to those with profound anemia and advanced hemodynamic instability, provided modern mechanical support and hematologic optimization are available. Future research should focus on multicenter prospective protocols and comparative outcomes studies to solidify evidence-based best practices.

## Data Availability

No new data were created or analyzed in this study. Data sharing is not applicable to this article.
